# Accounting for Modeling Errors and Inherent Structural Variability through a Hierarchical Bayesian Model Updating Approach: An Overview

**DOI:** 10.3390/s20143874

**Published:** 2020-07-11

**Authors:** Mingming Song, Iman Behmanesh, Babak Moaveni, Costas Papadimitriou

**Affiliations:** 1 Department of Civil and Environmental Engineering, Tufts University, Medford, MA 02155, USA; mingming.song@tufts.edu; 2 Structural Engineering Division, Simpson Gumpetz & Heger, New York, NY 10018, USA; ibehmanesh@sgh.com; 3 Department of Mechanical Engineering, University of Thessaly, 38334 Volos, Greece; costasp@uth.gr

**Keywords:** hierarchical Bayesian modeling, model updating, modeling errors quantification, reinforced concrete buildings, structural identification, uncertainty quantification

## Abstract

Mechanics-based dynamic models are commonly used in the design and performance assessment of structural systems, and their accuracy can be improved by integrating models with measured data. This paper provides an overview of hierarchical Bayesian model updating which has been recently developed for probabilistic integration of models with measured data, while accounting for different sources of uncertainties and modeling errors. The proposed hierarchical Bayesian framework allows one to explicitly account for pertinent sources of variability such as ambient temperatures and/or excitation amplitudes, as well as modeling errors, and therefore yields more realistic predictions. The paper reports observations from applications of hierarchical approach to three full-scale civil structural systems, namely (1) a footbridge, (2) a 10-story reinforced concrete (RC) building, and (3) a damaged 2-story RC building. The first application highlights the capability of accounting for temperature effects within the hierarchical framework, while the second application underlines the effects of considering bias for prediction error. Finally, the third application considers the effects of excitation amplitude on structural response. The findings underline the importance and capabilities of the hierarchical Bayesian framework for structural identification. Discussions of its advantages and performance over classical deterministic and Bayesian model updating methods are provided.

## 1. Introduction

With the availability of cheaper sensors and advanced computational tools, the inference of models from data is receiving more attention in different engineering applications, including structural engineering [1,2,3,4]. Among the different numerical models, mechanics-based models such as finite element (FE) models are frequently utilized in structural design, response prediction to future loading, damage identification and structural health monitoring. The prediction accuracy of models can be improved by integrating measurements from real structural systems with numerical models through model updating. In this process, certain structural parameters such as material properties are tuned so that model predictions match the observed data. This process is referred to as model updating, model calibration, system identification, model inversion, model inference, or digital twinning, in the literature [1,2,3,4]. Model updating is traditionally performed through an optimization process, referred to as ‘deterministic approach’ here, or through Bayesian inference. In the deterministic approach, structural parameters are updated by minimizing a user-defined objective function, which consists of the discrepancies between model-predictions and field measurements [1]. This approach is easy to implement and often not computationally demanding if local optimization algorithms are used [5,6]. Therefore, a large number of deterministic model updating applications can be found in the literature, on a variety of laboratory or full-scale civil structures [1,2,3,4,6,7,8,9,10,11,12,13,14,15,16,17,18]. Bayesian model updating methods have received more interest in structural engineering applications over the last two decades [19]. In addition to the optimal values of updating parameters (as most probable), Bayesian methods also quantify the estimation uncertainty of parameters which can be propagated into response predictions. A joint posterior probability distribution of structural parameters is obtained using Bayesian inference by combining the prior knowledge of parameters and the likelihood of measurements. The *maximum a posteriori* (MAP) solution (the peak of posterior distribution) represents the optimal or most probable solution. The variances of marginal probability distributions of updating parameters represent their estimation uncertainties. The joint posterior distribution of parameters can be estimated analytically through an asymptotic approximation method [20], or numerically through stochastic sampling methods [21,22,23]. The response can then be predicted probabilistically using the updated model, by propagating the parameters uncertainty. Several applications of Bayesian model updating on numerical [19,24,25,26,27,28,29,30], laboratory [31] and real-world [32,33,34,35] structures can be found in the literature.

The uncertainties/variabilities concerning model updating can be categorized into three groups: (1) measurement noise, which includes sensor or cable noise for time history response, as well as estimation errors in data feature extraction, e.g., modal identification errors; (2) variability of effective structural properties (referred to as ‘inherent variability’ of structural parameters), such as mass, stiffness, or boundary conditions due to changing ambient and environmental conditions, such as temperature, wind and traffic loads, human activity, humidity, or excitation level; and (3) modeling errors in the numerical model due to, for example, discretization in FE models, unmodeled non-structural components, modeling simplifications and assumptions with respect to linearity, material constitutive model, connections, and boundary conditions. Modeling errors are often the most significant and influential source of uncertainty in modeling, model updating and response predictions, especially for civil structures, due to their large-scale size and complexity [29,36,37]. 

While the deterministic model updating methods can consider the effects of measurement noise/uncertainty by integrating residual weights in the objective function, these methods cannot quantify the estimation uncertainty of updating parameters. The classical Bayesian model updating methods lump the effects of all uncertainties into the error function (i.e., prediction error) and often consider them as a zero-mean Gaussian white noise in the likelihood function. Some work have been done to study the effects of spatial and temporal error correlation on Bayesian inference and the identifiability of joint parameter and error function [38,39], however, zero-mean Gaussian error function is still commonly adopted in practice [19,20]. In such Bayesian approaches, the inherent structural variability and modeling errors are not explicitly accounted for or quantified, and the estimation uncertainty of updating parameters is only assumed to be caused by measurement noise. Therefore, estimation uncertainty monotonically decreases as more measurements are used in the likelihood function and converges to zero for adequately large amount of data. The underestimation and monotonically decreasing trend of parameter uncertainty, conditional on parameter being globally or locally identifiable [20], using the classical Bayesian methods have been demonstrated through a numerical study of a three-story shear building model [40]. This decreasing trend is described as ’noise mitigation’ in [41]. This implies that the classical Bayesian inference shall not be used for the quantification of model updating uncertainties unless the uncertainties are limited to measurement noise, which is often not the case in civil structural systems. However, both deterministic and Bayesian approaches are effective in finding the optimal structural parameters from measured data. Alternatively, non-probabilistic approaches, e.g., fuzzy set theory, can be employed to account for uncertainties in model updating [42], but inherent structural variability and modeling errors are not explicitly quantified in these methods either.

Hierarchical Bayesian model updating has been proposed to address the aforementioned shortcomings in estimation of realistic structural parameter uncertainty and quantification of modeling errors. Hierarchical Bayesian modeling [43] was first developed by mathematicians to estimate unknown parameters at higher levels which measurements were not directly related to, e.g., estimation of distribution parameters-mean and standard deviation-(referred to as hyperparameters) of structural parameters. In applications to civil structural systems, it is reasonable to model the structural components properties with an underlying joint probability distribution, to represent and account for aforementioned three types of uncertainties, (1) measurement noise, (2) the inherent variability due to changing ambient and environmental conditions, and (3) the effects of modeling errors, e.g., different nonlinear mechanisms activated in the real systems that have not been accounted for in the numerical model [44]. Therefore, in a hierarchical Bayesian framework, instead of directly estimating the structural parameters (e.g., stiffness), the hyperparameters (e.g., mean vector and covariance matrix of stiffness probability distribution) are estimated. This approach is suitable when multiple sets of measurements are available and each set of measurements is assumed to only contain information about one realization of the structural parameters (e.g., effective structural stiffness under different experimental conditions). Therefore, by constructing a hierarchical model, the distribution parameters (hyperparameters) of the structural parameters, which cannot be directly inferred from an individual set of measurements, are estimated from multiple realizations of the dynamic system. In this case, the effects of pertinent uncertainties can be considered and quantified by estimating structural hyperparameters to represent structural parameters variability. 

Moreover, the hierarchical framework provides an estimate for the distribution parameters of the error function. The error function, which represents the prediction error between model-predictions and field measurements, is assumed to follow a probability distribution characterized by its distribution parameters, e.g., mean vector and covariance matrix. The error function here represents the residual uncertainty between model-predictions and measurements after a portion of the total system uncertainties has been embedded in and accounted for by hyperparameters. Therefore, the error function captures the remaining prediction misfit which cannot be captured by the hyperparameter-characterized structural parameters variability. For comprehensive and robust probabilistic response predictions using the calibrated model, both parameter uncertainties (hyperparameters) and error function uncertainties (mean vector and covariance matrix of error function distribution) are included and propagated. 

Following a review of the hierarchical Bayesian model updating framework, the paper provides applications of the hierarchical Bayesian approach on three full-scale civil structures, where structural hyperparameters and error function are estimated. The first application considers a two-span footbridge at Tufts University campus in Medford, Massachusetts. The footbridge was instrumented with a continuous monitoring system including accelerometers and thermocouples. Three different updating scenarios are performed to underline the capabilities of the hierarchical approach to consider different subsets of available heterogeneous measurements, i.e., (i) only vibration data, (ii) vibration data and ambient temperatures, or (iii) vibration data, ambient temperatures, and excitation amplitudes. The second case study is a 10-story reinforced concrete (RC) building in Utica, New York, instrumented with accelerometers. This application investigates the effects of the error function distribution format (zero-mean vs. non-zero-mean) on response predictions, and discusses the shortcomings of using a tightly fitted model for extrapolation outside the data range, i.e., error function distribution changes outside the observed data range. The third case study is a two-story RC building in El Centro, California, which was excessively damaged by past earthquakes. In this application, the structural stiffness hyperparameters are assumed to be function of loading amplitudes. Dynamic response of the building to a shaker excitation is predicted when effect of loading amplitudes is or is not considered.

## 2. Uncertainty Quantification and Propagation through Hierarchical Bayesian Modeling

### 2.1. Sources of Uncertainty

#### 2.1.1. Measurement Noise 

In this paper, measurement noise is generalized to denote any errors/discrepancy between available data features used for model updating and their true counterparts from the actual structure. Therefore, measurement noise can either be (1) cable or sensor noise when time history measurements are used for model updating, e.g., acceleration, velocity, displacement, and strain, etc., or (2) data feature extraction errors, e.g., modal identification errors when modal parameters (natural frequencies and mode shapes) are used for model updating, as have been done in the three reviewed applications. Modal identification errors are often caused by linear model assumption, viscous damping assumption, experimental variability, data processing errors and analyst’s subjective errors [45]. It is worth noting that modal identification errors can alternatively be considered as a type of modeling errors, e.g., due to linear model assumption considered in the identification process, while the structure is inherently nonlinear. However, in this paper, we consider this as measurement noise, because it has similar effects to sensor noise when modal parameters are used for model updating. The term ‘modeling errors’ in this paper particularly refers to the errors/uncertainty in the numerical model. A hierarchical Bayesian modal analysis framework has been recently developed to quantify the variability of modal properties due to modal analysis errors [45]. It has been demonstrated that modal properties vary over datasets, even if ambient and environmental conditions over different datasets are unchanged. This variability is mainly due to the linear model assumption used in modal analysis, and to a lesser extent, due to the data processing errors.

#### 2.1.2. Changing Ambient and Environmental Conditions 

An important source of variability on effective structural properties, which is relatively unique to civil structural systems, is the changing ambient and environmental conditions. Past studies have shown that effective structural properties, such as stiffness, mass or boundary conditions, can vary due to the changes in ambient conditions (e.g., wind load, traffic load, occupancy, and human activity), as well as environmental conditions (e.g., temperature, temperature gradient, humidity, and rainfall) [46,47,48,49,50]. For example, structural mass property changes due to occupancy in a building, traffic on a bridge, or precipitation. The freezing of a bridge deck can contribute to the structural stiffness significantly, as reported in a year-long monitoring of the Z-24 bridge in Switzerland [46]. Some experimental studies on the Alamosa Canyon Bridge also showed that temperature differentials across the concrete deck had a strong influence on the bridge natural frequency and its stiffness (first mode frequency varied approximately 5% during a 24 h period) [47], and evident reductions in natural frequencies were observed because of the increase of bridge mass, due to rainfall [48]. Clinton et al. [49] studied the long-term natural frequencies of the Millikan Library and the Broad Center, and observed significant variations in their natural frequencies when weather conditions change, e.g., heavy rain decreased fundamental frequencies by up to 3%, and high temperatures raised all natural frequencies by 1–2%, while strong winds decreased the natural frequencies by up to 3%. Moser and Moaveni [50] also reported a significant increase in natural frequencies of the Dowling Hall footbridge, when temperatures go below the freezing point. They attribute this to stiffer boundary conditions (soil freezing) and deck hardening. This type of variability in structural properties is referred to as ‘inherent variability’ in this study to be distinguished from the other types of uncertainties: modeling errors and measurement noise. For mechanical systems, other factors such as manufacturing variability can contribute to the inherent structural variability as well [51].

#### 2.1.3. Modeling Errors 

Modeling errors are often the most critical and influential source of uncertainty in modeling, model updating and response predictions, especially for civil structures, due to their complexity and large-scale size [37,52]. Different modeling assumptions and simplifications can introduce errors in the numerical models, including, but not limited to, structural linearity assumption, boundary conditions simplification, unmodeled non-structural components, material property uncertainty, discretization, geometry errors, and connection simplification. Sedehi et al. [44,53] have demonstrated that different model updating results can be obtained using different sets of measurements (or different windows of data from a single experiment), even when all experiment factors are kept constant, including ambient and environmental conditions, and excitation characteristics, such as frequency content and intensity. In these applications, time history measurements were used with negligible measurement noise; therefore, the observed parameter estimation variability is attributed to the effects of modeling errors of the numerical model used for model updating.

### 2.2. Hierarchical Bayesian Model Updating

#### 2.2.1. Hyperparameters 

Due to the uncertainties concerning model updating, i.e., measurement noise, the inherent variability of structural properties and modeling errors, it is reasonable to consider unknown updating structural parameters as random variables with underlying probability distributions, to be estimated in the hierarchical Bayesian approach. It is worth noting that, in the classical Bayesian inference, true values of updating structural parameters (e.g., stiffness) are not assumed to follow an underlying probability distribution, but some unknown constant values. The updating structural parameters are estimated to follow the joint posterior probability density function (PDF) in the classical Bayesian approach, from combining the information from prior PDF and likelihood function. However, the estimation uncertainty of parameters observed in the posterior PDF is only caused by measurement noise, and represents the confidence of parameters estimation. Therefore, the posterior PDF will shrink to a Dirac delta function, with increasing data points used in the likelihood function if the inverse problem is globally or at least locally identifiable [40]. In the reviewed applications of hierarchical Bayesian modeling in this paper, structural parameters θ, i.e., effective stiffness of different structural components, are considered to follow a priori distribution (e.g., Gaussian), characterized by unknown distribution parameters referred to as ‘hyperparameters’, e.g., mean vector $\mu_{\theta }$ and covariance matrix $\Sigma_{\theta }$ of the Gaussian distribution. Then, the hyperparameters are directly updated in the hierarchical framework, which govern the structural parameters variability through the assumed distribution. Note, that since hyperparameters are the updating parameters in this framework, posterior marginal distributions of hyperparameters quantify their estimation uncertainties similar to the estimation uncertainty of structural parameters in the classical Bayesian method. These estimation uncertainties are reduced by feeding more datasets, but their MAP estimates (most probable values) converge to constant values with an adequate number of data points. Therefore, the structural parameters variability is not affected by the number of data points used and represents the system uncertainty.

In the hierarchical approach, alternative distribution models, such as lognormal or Gamma distributions, can be assumed for updating structural parameters. However, in the reviewed applications, truncated (to be positive) Gaussian distribution is chosen for stiffness parameters due to the maximum entropy theory, i.e., the Gaussian distribution provides the maximum uncertainty, given that the mean and variance of the distribution are bounded. Stiffness parameters are the most common updating structural parameters in the literature, but other parameters, such as mass or geometric properties, can also be considered in a similar manner [34].

#### 2.2.2. Error Function 

Similar to classical model updating methods, an error function is defined to represent the misfit between model-predictions and the measurements. The error function is based on the available measured data, e.g., modal properties or time history measurements. Common forms of error functions using natural frequencies and mode shapes are defined by Equations (1) and (2).

(1) $$e_{\lambda_{tm}}=\frac{{\tilde{\lambda }}_{tm}-\lambda_{m}(\theta_{t})}{\lambda_{m}(\theta_{t})},$$

(2) $$e_{\Phi_{tm}}=\frac{{\tilde{\Phi }}_{tm}}{\left\|{\tilde{\Phi }}_{tm}\right\|}-a_{tm}\frac{\Gamma \Phi_{m}(\theta_{t})}{\left\|\Gamma \Phi_{m}(\theta_{t})\right\|},\;\mathrm{with}\;a_{tm}=\frac{{\tilde{\Phi }}_{tm}^{T}\Gamma \Phi_{m}(\theta_{t})}{\left\|{\tilde{\Phi }}_{tm}\right\|\left\|\Gamma \Phi_{m}(\theta_{t})\right\|}$$

where $e_{\lambda_{tm}}$ and $e_{\Phi_{tm}}$ are the error functions of natural frequencies and mode shapes for mode *m*, respectively. $\theta_{t}$ is the structural parameter at test or dataset *t*. ${\tilde{\lambda }}_{tm}$ and ${\tilde{\Phi }}_{tm}$ are the identified eigenvalues (square of circular natural frequency in rad/s) and mode shapes, and $\lambda_{m}(\theta_{t})$ and $\Phi_{m}(\theta_{t})$ are their model-predicted counterparts. Γ is the selection matrix which maps $\Phi_{m}(\theta_{t})$ to ${\tilde{\Phi }}_{tm}$. $a_{tm}$ is a scaling factor which makes the identified and model-predicted mode shapes comparable. Error functions can also be defined using time history measurements (e.g. acceleration, displacement or strain) if the excitation loads are known, as done in [44]:

(3) $$e(k,\theta_{t})=y(k)-\Gamma x(k,\theta_{t}),$$

where **y** refers to time history measurements, **x** is model-predicted counterparts and *k* is the time index. 

In the hierarchical Bayesian framework, the error function is considered as a random variable, following a priori distribution (similar to structural parameters) with unknown distribution parameters. In this paper, Gaussian distributions are considered to maximize the information entropy, but other distributions can be considered. The mean vector $\mu_{e}$ and covariance matrix $\Sigma_{e}$ of the error function are estimated, along with the structural hyperparameters $\mu_{\theta }$ and $\Sigma_{\theta }$.

#### 2.2.3. Model Updating Process 

**Figure 1:**
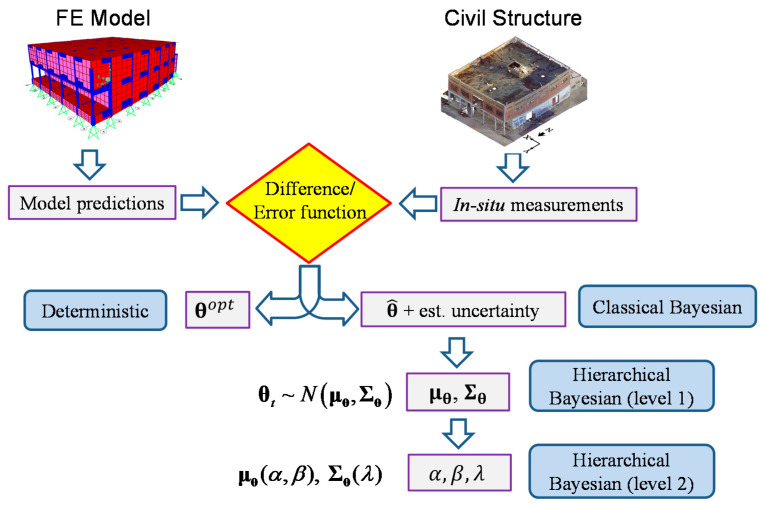
Comparison of deterministic, classical Bayesian and hierarchical Bayesian methods: In deterministic approach, optimal structural parameter $\theta^{opt}$ is estimated; In classical Bayesian methods, most probable structural parameter $\hat{\theta }$ is estimated with its estimation uncertainty; At hierarchical level 1, $\mu_{\theta }$ and $\Sigma_{\theta }$ are estimated, and at hierarchical level 2, *α*, *β*, and *λ* are estimated

In the hierarchical approach, the updating structural parameters Θ, stiffness parameters used in this paper, consists of vector of effective stiffness for different structural components corresponding to different individual test *t,* $\Theta =\left\{\theta_{t},\;t=1,\cdots ,N_{t}\right\}$, and $N_{t}$ refers to the total number of datasets/experiments. Stiffness hyperparameters are denoted by mean vector $\mu_{\theta }\left(\alpha ,\beta \right)$ and covariance matrix $\Sigma_{\theta }(\lambda )$, while the error function distribution is characterized by mean vector $\mu_{e}$ and covariance matrix $\Sigma_{e}$. The hyperparameters $\mu_{\theta }\left(\alpha ,\beta \right)$ and $\Sigma_{\theta }\left(\lambda \right)$ can be assumed to be functions of higher-level variables (optional higher-level hyperparameters) *α*, *β*, *λ*, or even more variables, as needed. These higher-level hyperparameters can represent coefficients of a model between stiffness mean (or covariance) and other influential factors of interest, such as ambient temperature and excitation level. When such relationship models are considered, then these coefficients variables *α*, *β*, and *λ* will be directly estimated, and hyperparameters $\mu_{\theta }$ and $\Sigma_{\theta }$ are computed using these coefficients. In the absence of such relationship, $\mu_{\theta }$ and $\Sigma_{\theta }$ will be estimated directly. It is worth noting that by constructing the relationships between hyperparameters and other influential factors, the estimated stiffness variability (covariance matrix $\Sigma_{\theta }$) will be reduced compared to when estimating $\mu_{\theta }$ and $\Sigma_{\theta }$ directly without considering this relationship. This is due to the fact that these influential factors account for different sources of uncertainty, and by including them in the hierarchical model, more accurate predictions with tighter confidence bounds are obtained from the updated model. The hierarchical Bayesian framework has the capability to model several levels/hierarchies of correlated information, and therefore reduce the estimated variability of structural parameters by including extra levels of information. A schematic comparison graph between the hierarchical Bayesian approach with traditional deterministic and Bayesian methods is shown in Figure 1.

The joint posterior PDF of the updating parameters conditioned on all datasets D ($D=\left\{D_{t},\;t=1,\cdots ,N_{t}\right\}$) is shown below, by applying the Bayes’ theorem:

(4) $$p\left(\Theta ,\mu_{\theta }\left(\alpha ,\beta \right),\Sigma_{\theta }\left(\lambda \right),\mu_{e},\Sigma_{e}|D\right)\propto p\left(D|\Theta ,\mu_{\theta }\left(\alpha ,\beta \right),\Sigma_{\theta }\left(\lambda \right),\mu_{e},\Sigma_{e}\right)p\left(\Theta ,\mu_{\theta }\left(\alpha ,\beta \right),\Sigma_{\theta }\left(\lambda \right),\mu_{e},\Sigma_{e}\right)$$

(5) $$\propto p\left(D|\Theta ,\mu_{e},\Sigma_{e}\right)p\left(\Theta |\mu_{\theta }\left(\alpha ,\beta \right),\Sigma_{\theta }\left(\lambda \right),\mu_{e},\Sigma_{e}\right)p\left(\mu_{\theta }\left(\alpha ,\beta \right),\Sigma_{\theta }\left(\lambda \right),\mu_{e},\Sigma_{e}\right),$$

(6) $$\propto p\left(\mu_{\theta }\left(\alpha ,\beta \right)\right)p\left(\Sigma_{\theta }\left(\lambda \right)\right)p\left(\mu_{e}\right)p\left(\Sigma_{e}\right)\prod_{t=1}^{N_{t}}p\left(D_{t}|\theta_{t},\mu_{e},\Sigma_{e}\right)p\left(\theta_{t}|\mu_{\theta }\left(\alpha ,\beta \right),\Sigma_{\theta }\left(\lambda \right)\right),$$

in which different datasets are assumed to be statistically independent. In Equation (5), the conditioning on $\mu_{\theta }\left(\alpha ,\beta \right)$ and $\Sigma_{\theta }\left(\lambda \right)$ is dropped from $p\left(D|\Theta ,\mu_{\theta }\left(\alpha ,\beta \right),\Sigma_{\theta }\left(\lambda \right),\mu_{e},\Sigma_{e}\right)$ since measurements only directly depend on Θ which is controlled by hyperparameters. In Equation (6), the conditioning on $\mu_{e}$ and $\Sigma_{e}$ is dropped from $p\left(\Theta |\mu_{\theta }\left(\alpha ,\beta \right),\Sigma_{\theta }\left(\lambda \right),\mu_{e},\Sigma_{e}\right)$, since Θ only depends on stiffness hyperparameters. In this formulation, $p\left(D|\Theta ,\mu_{\theta }\left(\alpha ,\beta \right),\Sigma_{\theta }\left(\lambda \right),\mu_{e},\Sigma_{e}\right)$ is referred to as the ‘likelihood function’, which denotes the PDF of obtaining measurements D given updating parameters, and $p\left(\mu_{\theta }\left(\alpha ,\beta \right),\Sigma_{\theta }\left(\lambda \right),\mu_{e},\Sigma_{e}\right)$ is called the ‘prior distribution’, which is chosen by the practitioner based on prior information or engineering judgement. The updating parameters have been assumed to be independent in the joint prior distribution, and therefore, individual prior distributions, $p\left(\mu_{\theta }\left(\alpha ,\beta \right)\right)$, $p\left(\Sigma_{\theta }\left(\lambda \right)\right)$, $p\left(\mu_{e}\right)$, and $p\left(\Sigma_{e}\right)$ are used in Equation (6).

The likelihood function $p\left(D_{t}|\theta_{t},\mu_{e},\Sigma_{e}\right)$ follows the distribution of error functions, and $p\left(\theta_{t}|\mu_{\theta }\left(\alpha ,\beta \right),\Sigma_{\theta }\left(\lambda \right)\right)$ is defined by the assumed distribution of stiffness parameters. In this paper, joint Gaussian distributions are considered for both. The prior distributions, $p\left(\mu_{\theta }\left(\alpha ,\beta \right)\right)$, $p\left(\Sigma_{\theta }\left(\lambda \right)\right)$, $p\left(\mu_{e}\right)$ and $p\left(\Sigma_{e}\right)$ can be chosen to reflect the prior knowledge of these parameters or engineering judgment, e.g., information from structural material tests can be considered in determining the prior distributions of hyperparameters $p\left(\mu_{\theta }\left(\alpha ,\beta \right)\right)$ and $p\left(\Sigma_{\theta }\left(\lambda \right)\right)$. In the following three applications, specific priors are used which are defined as ‘conjugate priors’ in the Bayesian theory [43]. The selection of conjugate priors has the advantage of simplifying the mathematical derivation and computation of the posterior PDFs, since posterior distributions belong to the same family of distributions as the prior one. For example, in Application 1, gamma distribution is used for $\Sigma_{\theta }$, and in Application 3, inverse-Wishart distribution is selected for $\Sigma_{\theta }$. However, use of conjugate priors is not a requirement and other distributions can be alternatively used. 

To reduce the number of independent updating parameters, the covariance matrix $\Sigma_{\theta }$ can be assumed to be diagonal, i.e., only diagonal terms need to be estimated, as has been done in past studies [40,54,55,56]. This assumption ignores the stiffness correlations of different structural components. Alternatively, a full covariance matrix $\Sigma_{\theta }$ can be estimated to characterize the stiffness correlations in off-diagonal terms, as it is done in some recent studies [53,57,58]. The error function distribution is often assumed to be zero-mean, i.e., $\mu_{e}=0$, which neglects error bias and causes an inflated estimation of error covariance $\Sigma_{e}$ in applications with error bias. The choice of $\mu_{e}$ depends on the specific application of whether an error function bias is observed, as shown in Application 2. The error covariance matrix $\Sigma_{e}$ is often assumed to be a diagonal matrix which ignores the error correlations (similar to $\Sigma_{\theta }$). This is a common simplification and often a reasonable assumption, and its accuracy depends on the specific application and available data. Other options are available to reduce independent updating variables in the error covariance matrix, including the use of the covariance matrix function, which considers the exponential relationship for covariance components [59,60]. The full error covariance matrix can also be estimated if needed, demanding a higher computational effort.

By substituting the likelihood functions and prior distributions into Equation (6), the joint posterior PDF can be obtained. This is often a complicated distribution, and it is challenging to evaluate it analytically. Therefore, numerical methods are often employed to estimate the joint and marginal posterior distributions. Note that analytical asymptotic approximate solutions for marginal distributions of hyperparameters can be obtained when a large number of data points are available in each dataset as done by Ballesteros et al. [51] using natural frequencies, and Sedehi et al. [44,53] using time history measurements.

### 2.3. Probabilistic Response Prediction

Introducing structural parameters distribution governed by hyperparameters embeds uncertainties into the structural parameters by providing variability and flexibility to the numerical model to represent the measured data. As discussed before, realistic parameters uncertainty is obtained through hyperparameters in the hierarchical framework, while the parameters uncertainty in classical Bayesian inference is significantly underestimated, since it only accounts for estimation uncertainty which decreases monotonically with more data. The error function in the hierarchical approach represents the remaining uncertainty between model-predictions and measurements after a portion of the uncertainties has been embedded and accounted for by hyperparameters. Therefore, it captures the remaining prediction misfit, which cannot be captured by the hyperparameter-characterized structural parameters variability. As a result, the error function term in the hierarchical Bayesian method is smaller (often significantly smaller) than the one obtained using classical Bayesian methods, since the hierarchical method only assigns the residual prediction errors to the error function, while classical Bayesian methods lump all sources of uncertainties into the error function. 

Note, that in the hierarchical approach, there is a compensation effect between structural parameters variability $\Sigma_{\theta }$ and error function covariance $\Sigma_{e}$, and the portion of total uncertainty that is assigned to each term depends on specific applications and factors such as model accuracy, plausibility of the assumed structural parameters distribution in the view of measurements, dimension of parameter space (a larger dimension of hyperparameters provides a higher flexibility of model predictions). For example, in Applications 1-3 of this paper, larger uncertainties are assigned to the error functions, and therefore error function uncertainties have to be propagated into probabilistic response predictions, while in studies by Sedehi et al. [44,53], uncertainties of structural parameters dominate the response predictions, since the numerical model is capable of representing the small laboratory 3-story structure well. For large-scale civil structure applications, a larger portion of uncertainty is often assigned to error function term, due to significant modeling errors.

**Figure 2:**
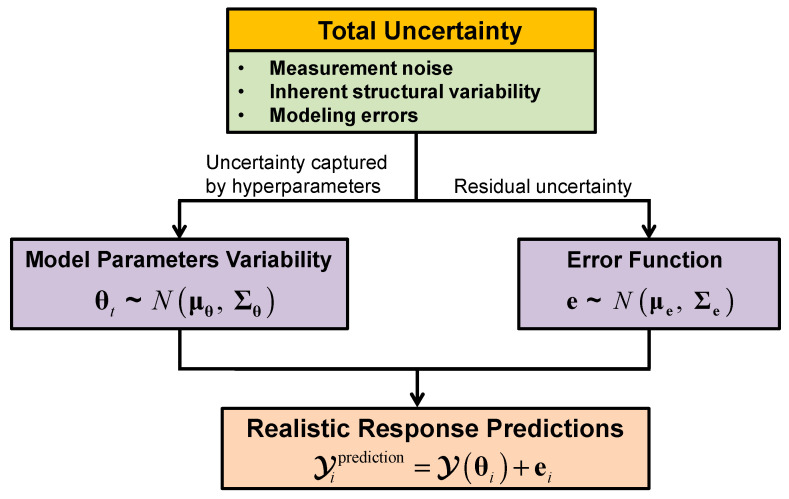
Uncertainties propagation into response predictions.

An important feature of hierarchical Bayesian model updating is the capability to provide accurate and realistic confidence bounds on response predictions, by propagating the estimated structural parameters variability and error function uncertainty, as shown in Figure 2. When response predictions of unobserved quantities (where error function estimate is not available) are required, two strategies can be adopted: (1) only consider and propagate the structural parameters variability into response predictions when error function is relatively small and negligible [44,53]; (2) extend the error function to unobserved degrees of freedom (DOFs) as proposed by Song et al. [58] and validated through a numerical study. As mentioned previously, in most civil structure applications, error function uncertainty must be included in model-predictions to fully capture the measurement variability.

Once the stiffness hyperparameters ($\mu_{\theta }$ and $\Sigma_{\theta }$) and error function distribution parameters ($\mu_{e}$ and $\Sigma_{e}$) are estimated, probabilistic model predictions can be performed using Monte Carlo simulation. Let $\theta_{i}∼N\left(\mu_{\theta },\Sigma_{\theta }\right)$ and $e_{i}∼N\left(\mu_{e},\Sigma_{e}\right)$ for i=1, ⋯, n denote *n* independent samples generated from the stiffness and error distribution, then *n* independent response predictions can be computed as:

(7) $$Y_{i}^{\mathrm{prediction}}=Y\left(\theta_{i}\right)+e_{i},$$

in which $Y\left(\theta_{i}\right)$ refers to the response predictions of the FE model with stiffness parameter $\theta_{i}$. When only structural parameters variability is propagated into response predictions, the error function term $e_{i}$ in Equation (7) is neglected. The predictions can be performed for any type of structural response depending on the error function used, e.g., modal properties (natural frequencies and mode shapes) or time history response (acceleration, velocity, displacement, and strain). If modal properties are used in model updating, then the modal superposition principle can be utilized to obtain the time history response predictions, as shown in Application 3.

### 2.4. Numerical Methods for Estimating Posterior Distributions

#### 2.4.1. Sampling Approach

The Markov chain Monte Carlo (MCMC) sampling technique has been shown to be an efficient numerical tool for sampling the posterior probability distribution using Bayesian inference. Different MCMC algorithms have been developed, including the Metropolis-Hastings (MH) algorithm [21], adaptive MH algorithms [22,61], transitional MCMC [23], and Gibbs sampling [62]. For the posterior PDF in Equation (6), sampling techniques like MH or adaptive MH can be inefficient since the acceptance rate of samples becomes too small, yielding very few distinct samples even after many steps. This is due to the high dimension of sampling space as the dimension of Θ increases with datasets number $N_{t}$, and dimensions of $\mu_{e}$ and $\Sigma_{e}$ increase with number of sensors employed. The appropriate selection and tuning of the proposal distribution might improve the acceptance rate in some cases, but it becomes more challenging for sampling very peaked posterior PDFs [23,30]. Gibbs sampling is shown to be computationally efficient for this task, as it transforms the joint posterior PDF into individual conditional PDFs where the samples are taken from, as shown in [40,57,58,62]. Since conditional PDFs have smaller dimensions, the sampling acceptance rate can be adjusted to the desired range. For the sampling of the conditional PDFs, other MCMC algorithms can also be applied as needed, for example, the MH within Gibbs sampling algorithm is applied in two recent applications by the authors [57,58].

#### 2.4.2. Simplified Approach for Estimating Map Values 

Gibbs sampling is more efficient to generate samples of high-dimension posterior PDF by sampling conditional distributions instead of joint posterior distribution. However, it can still be computationally prohibitive for applications to complex dynamic systems, especially when detailed FE models with large DOFs are employed. This is because the sampling process requires evaluating likelihood function at each step forward, which demands computing the response of FE models. A simplified and computationally efficient approach is proposed to estimate the MAP values of updating parameters through an optimization algorithm [56]. The simplified approach could not provide the estimation uncertainty of updating parameters. However, the estimation uncertainty of parameters decreases monotonically with increasing datasets used in the updating process and become negligible with adequate amount of data. It is worth remembering that since the hyperparameters (mean vector and covariance matrix of structural parameters) are updated, their estimation uncertainties are reduced using more datasets, but their MAP will converge to constant values representing the stiffness probability distribution. Therefore, realistic estimates of stiffness variability can be provided by the proposed simplified numerical approach within the hierarchical framework. However, in the classical Bayesian inference, even complete sampling of the posterior PDF can only represent the uncertainty due to measurement noise, which is monotonically reduced using more datasets. A combination of the simplified MAP-only approach with the Gibbs sampling technique can be implemented to promote the computational efficiency and provide parameters estimation uncertainty, as shown in Application 3. Alternatively, surrogate models can be employed to replace FE models in the sampling process to reduce the computation burden of evaluating the model-predicted response [59,63].

## 3. Applications to Three Full-Scale Civil Structures

### 3.1. Application 1: Footbridge at Tufts University Campus

#### 3.1.1. Test Structure and Measured Data

**Figure 3:**
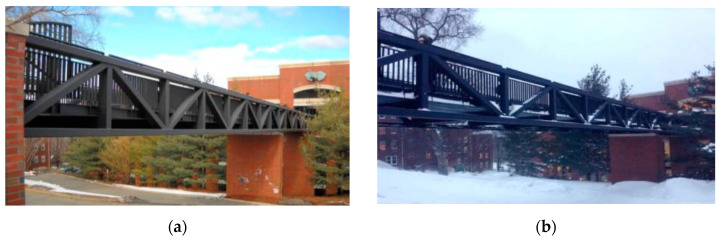
South-east view of Dowling Hall footbridge in (**a**) above-freezing, and (**b**) below-freezing conditions [54].

**Figure 4:**
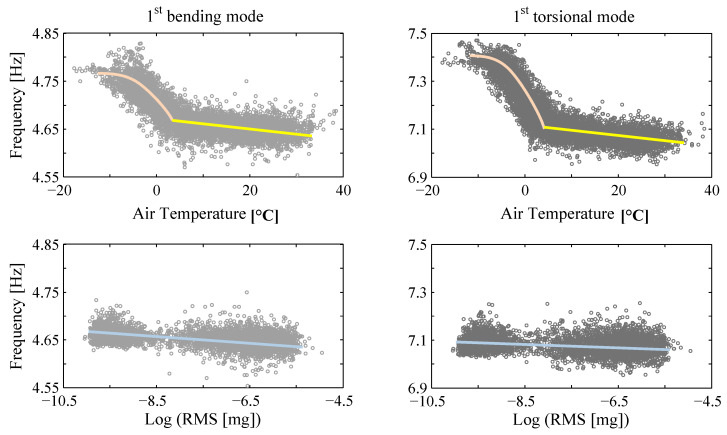
Effects of temperatures and excitation levels on identified natural frequencies of Dowling Hall footbridge.

The first case study considers the Dowling Hall footbridge located at Tufts University campus in Medford, Massachusetts [54]. Figure 3 shows the south-east view of the footbridge at above-freezing and below-freezing conditions. The footbridge is a continuous two-span (22 m each span) steel frame bridge with a concrete deck (width of 3.9 m). The footbridge was instrumented with a continuous monitoring system, including 12 accelerometers located along the two sides of the bridge deck measuring vertical vibration, and 10 thermocouples measuring the air, steel and concrete temperatures. The monitoring system recorded bridge ambient vibration for five minutes at the beginning of every hour. From each 5-min window of data, a set of modal parameters of the footbridge (natural frequencies and mode shapes) are extracted using the Stochastic Subspace Identification [64] method. Six vibration modes of the bridge are identified, and a total of 8721 datasets of modal parameters are extracted from the vibration data recorded from January 2010 to March 2012. Among all datasets, 1824 sets correspond to temperatures below the freezing point. The identified natural frequencies are significantly affected by ambient temperatures, and moderately affected by the excitation levels, as shown in Figure 4. The natural frequencies increase evidently as the temperatures drop, especially when temperatures go below the freezing point. A smaller, but non-negligible, correlation between natural frequencies and excitation levels is also observed, as demonstrated in Figure 4. The experimental data of the Dowling Hall footbridge is available online as reported in the Supplementary Materials section at the end of this paper.

#### 3.1.2. Hierarchical Bayesian Modeling at Different Information Levels

A linear FE model of the footbridge was created in Matlab [65]. Effective Young’s modulus of the concrete deck is selected as the only updating model parameter θ in this study. Therefore, hyperparameters $\mu_{\theta }$ and $\sigma_{\theta }^{2}$ are also scalar, which has reduced the computation burden of model updating. Hierarchical Bayesian model updating is implemented using different levels of information, namely (i) only modal parameters, (ii) modal parameters and temperatures, and (iii) modal parameters, temperatures, and excitation levels.

*Information level 1: only modal parameters*

At this information level, the stiffness variability was characterized by hyperparameters $\mu_{\theta }$ and $\Sigma_{\theta }$, as $\theta_{t}∼N\left(\mu_{\theta },\Sigma_{\theta }\right)$, while the effects of temperature and excitation amplitude are not explicitly modeled. This is the basic scenario of hierarchical Bayesian inference, where stiffness is assumed to follow a normal distribution, with no explicit relationship between stiffness and measurable sources of uncertainty considered. 

*Information level 2: modal parameters and temperatures*

In this case, in addition to the identified modal parameters, measured temperatures are used in the updating process. A model to represent the relationship between stiffness hyperparameters and measured temperatures is considered in Equations (8) and (9):

(8) $$\mu_{\theta }\left(T_{t}\right)=Q+S\times T_{t}+R\times \left(1-\mathrm{erf}\left(\frac{T_{t}-ϒ}{\tau }\right)\right),$$

(9) $$\sigma_{\theta }^{2}\left(T_{t}\right)=\mu_{\theta }^{2}\left(T_{t}\right)\times \sigma^{2},$$

where *Q*, *S*, *R*, ϒ, τ and σ are higher-level hyperparameters, describing the stiffness-temperature model. Moreover, $T_{t}$ is the average temperature (over five minutes) recorded for dataset *t*. The function representing stiffness mean versus temperature (Equation (8)) consists of a straight line, i.e., the first two terms, to capture the trend for temperatures above freezing point, and a nonlinear term (the ‘erf’ function denotes Gauss error function) to model the trend for temperature near and below freezing point. The stiffness standard deviation is assumed to change linearly with stiffness mean resulting a constant coefficient-of-variation (CoV) σ. This is assumed because larger variabilities are observed for larger stiffness and lower temperatures. It is worth noting that these higher-level hyperparameters (*Q*, *S*, *R*, ϒ, τ and σ) will be updated in the hierarchical Bayesian framework instead of $\mu_{\theta }$ and $\sigma_{\theta }^{2}$, which are functions of these higher-level hyperparameters based on Equations (8) and (9).

*Information level 3: modal parameters, temperatures, and excitation amplitudes*

In this case, the effects of excitation amplitudes are included in modeling stiffness hyperparameters in addition to temperature measurements. The stiffness mean model is similar to that of information level 2, with an additional excitation amplitude term added to the model:

(10) $$\mu_{\theta }\left(T_{t}\right)=Q+S\times T_{t}+R\times \left(1-\mathrm{erf}\left(\frac{T_{t}-ϒ}{\tau }\right)\right)+Y\times \mathrm{Log}\left(\varepsilon_{t}\right),$$

In this equation, Y refers to a new higher-level hyperparameter for excitation levels $\varepsilon_{t}$, which is defined as the root-mean-square (RMS) of acceleration measurements at test *t*. The fourth term represents the linear trend observed in Figure 4. The stiffness variance model is assumed to be the same as Equation (9). 

For all three information levels, the error function is assumed to follow a zero-mean Gaussian distribution, with a diagonal covariance matrix parameterized by only one parameter $\sigma_{e}^{2}$. This simplification reduces the number of updating parameters, however, the diagonal components of estimated error covariance might be inflated or underestimated, due to the smearing effect among different natural frequencies and mode shape components. A more accurate error covariance estimation can be obtained by updating all diagonal terms, or even the full matrix. 

#### 3.1.3. Model Updating Results and Response Predictions 

**Figure 5:**
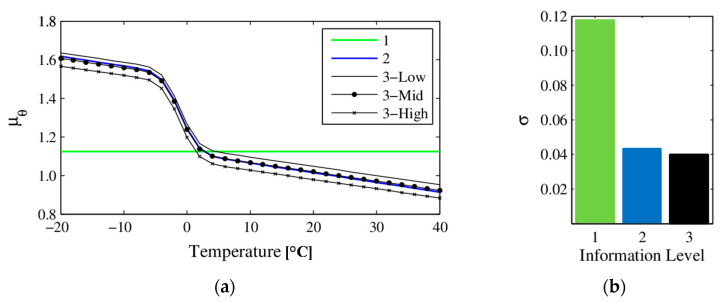
Estimated stiffness mean (**a**) and variance (shown as CoV) (**b**) at information levels 1, 2 and 3 [54].

The statistics (mean and standard deviation) of higher-level hyperparameters for information level 3 are reported in Table 1, which are evaluated from Gibbs sampling. It can be seen that, except for ϒ and τ, estimation uncertainties of all hyperparameters are very small. These two hyperparameters define the nonlinear transition of $\mu_{\theta }$ below and near freezing point. The estimated stiffness mean $\mu_{\theta }$ and CoV σ at information levels 1, 2 and 3 are shown in Figure 5. The mean is shown as a function of temperatures for information level 2, and at three different excitation amplitudes for information level 3, namely the minimum, average and maximum excitation amplitudes recorded. It can be seen that the stiffness mean stays constant at information level 1, since the effects of temperatures are not considered in this case; $\mu_{\theta }$ at information level 2 follows the considered model of nonlinear (below and around freezing) and linear (above freezing) trends similar to Figure 4; and $\mu_{\theta }$ shows a slight vertical shift due to different excitation amplitudes at information level 3. It is also observed that the stiffness variability (shown as CoV) is consistently reduced as more information is considered, especially when accounting for temperatures which provide significant reductions in CoV (from 0.12 to 0.04). 

**Figure 6:**
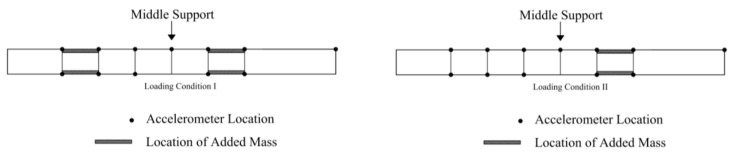
Loading scenarios I (left) and II (right) [54].

**Figure 7:**
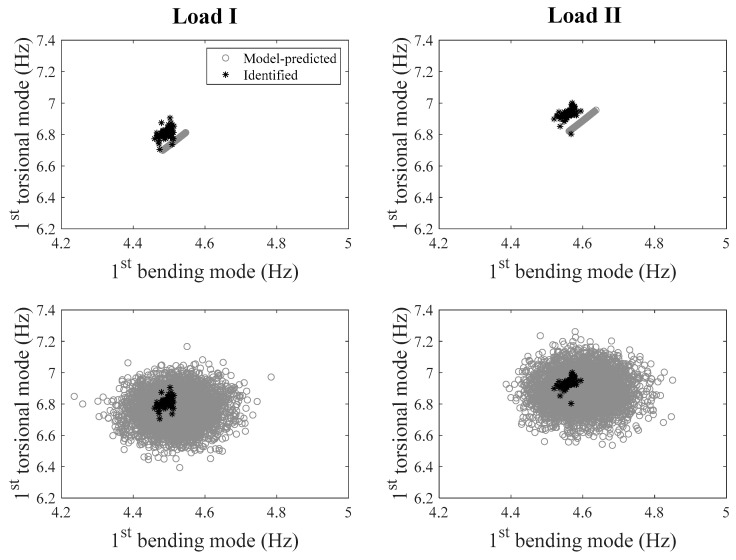
Comparison between model-predicted and identified natural frequencies in two loading scenarios (left column: loading scenario I; right column: loading scenario II), with/without accounting for error function (top row: without error function; bottom row: with error function).

To investigate the prediction capability of the calibrated model, natural frequencies of the bridge are predicted using the model in two loading scenarios. For the loading scenarios, the footbridge was divided into seven segments in the longitudinal direction, as shown in Figure 6. In loading scenario I, two of seven segments were loaded with concrete blocks (1.35 tons on each segment), and in scenario II, only one segment was loaded with concrete blocks of 1.35 tons. Concrete blocks were stacked on the sides of deck to allow pedestrian traffic. Model-predicted natural frequencies are obtained using the calibrated hierarchical Bayesian model at information level 3 (loading scenarios are also considered in the FE model), and compared with their experimentally identified counterparts after the loading scenarios were implemented on the bridge. Two different sets of predictions are provided with and without the propagation of error function uncertainty (which is considered as zero-mean Gaussian distribution), and are compared with their identified counterparts in Figure 7. It is observed that model predictions uncertainty is significantly underestimated compared to the identified natural frequencies when only structural parameters uncertainty is propagated (top row of Figure 7). The error function uncertainty has to be propagated into predictions to contain the identified values in both loading scenarios (bottom row of Figure 7). Therefore, a larger portion of uncertainties is captured by the error function rather than structural parameters due to large modeling errors and small flexibility of structural parameter distribution (only one stiffness parameter considered in the FE model). This indicates the importance of accounting for the effects of error function to provide realistic confidence bounds under future loading conditions. The confidence bounds are relatively wide compared to the variability of identified frequencies, due to the fact that zero-mean error function is used in this study, and the error variance is inflated to compensate for the existing bias (the offset between the centers of black and gray clouds in top row of Figure 7). The effects of zero-mean and non-zero-mean error function are studied and demonstrated in detail in Application 2 of this paper. It is worth reminding that the data from considered loading scenarios are not used in the updating process, and the calibrated model is ‘extrapolated’ for these predictions.

### 3.2. Application 2: Ten-Story RC Building in Utica, NY

#### 3.2.1. Test Structure and Measured Data

**Figure 8:**
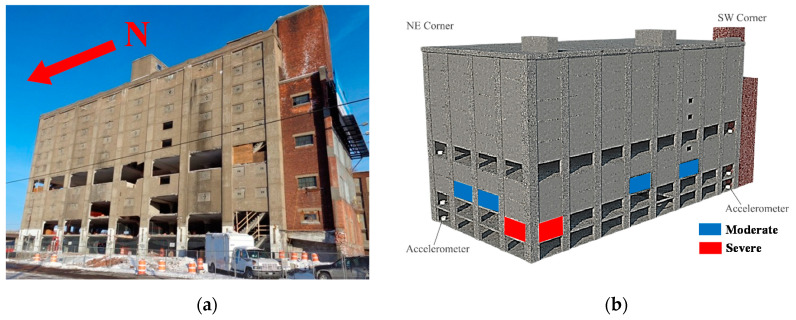
(**a**) South-west view of 10-story building; (**b**) location of removed walls for moderate and severe damage states.

The second application is a 10-story RC building in Utica, New York, as shown in Figure 8a [55]. The eight-bay by four-bay building consisted of a slab-column structural system with peripheral RC walls. Dynamic tests were performed on the structure before it was demolished for the construction of a new highway bridge. The building was instrumented with accelerometers at north-west and south-east corners on each floor, measuring the acceleration in two horizontal and the vertical directions resulting in 60 channels of acceleration measurements. The Natural Excitation Technique combined with the Eigensystem Realization Algorithm (NExT-ERA) [66,67] is used to extract modal parameters of the building using ambient vibration data collected over 32 h. Five modes of the structure are identified, and a total of 343 sets of modal parameters are obtained, with 50 of them used for model updating. The experimental data of this 10-sotry RC building is available online reported in Supplementary Materials at the end of this paper.

#### 3.2.2. Hierarchical Bayesian Model Updating with Zero-Mean and Non-Zero-Mean Error Function

**Figure 9:**
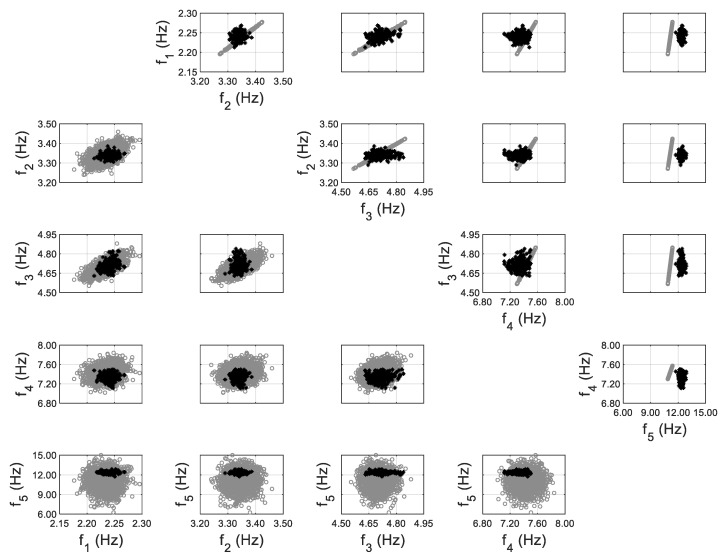
Model-predicted (gray) vs. identified (black) natural frequencies, using zero-mean error function (upper triangular subplots denote predictions without error function considered; lower triangular subplots denote predictions with propagation of error function).

A FE model of the structure is created using the Matlab toolbox FEDEASLab [68], and the floor slabs are assumed to be a rigid diaphragm. Two structural parameters are selected as the effective Young’s modulus of exterior walls and slabs. The error function is defined based on the first five modes of the structure identified using ambient data. The hierarchical Bayesian inference is first implemented by assuming the error function to follow a zero-mean Gaussian distribution, and the comparisons between the model-predicted and identified natural frequencies are shown in Figure 9, with and without the propagation of error function (only $\Sigma_{e}$ in this case). It can be seen that the identified frequencies fall out of the prediction regions when error function is not included (upper triangular subplots), while all identified values are within the high probability prediction regions after error function is propagated (lower triangular subplots). This is similar to the findings in Application 1, and underlines the importance of accounting for error function in response predictions, especially when a significant amount of uncertainty is retained in it. A significant bias is observed between predictions and identified values for the fifth mode (the last row of subplots), which results in a large prediction uncertainty for this mode, i.e., by assuming a zero-mean error function, the error variance is inflated to compensate for the bias. Therefore, model updating is performed again using a non-zero-mean error function for the fifth mode, which is evaluated as:

(11) $${\hat{\mu }}_{e}=\frac{1}{N_{t}}\sum_{t=1}^{N_{t}}{\hat{e}}_{t},$$

in which ${\hat{e}}_{t}$ denotes the evaluated error function with the updated stiffness ${\hat{\theta }}_{t}$ for dataset *t*.

**Figure 10:**
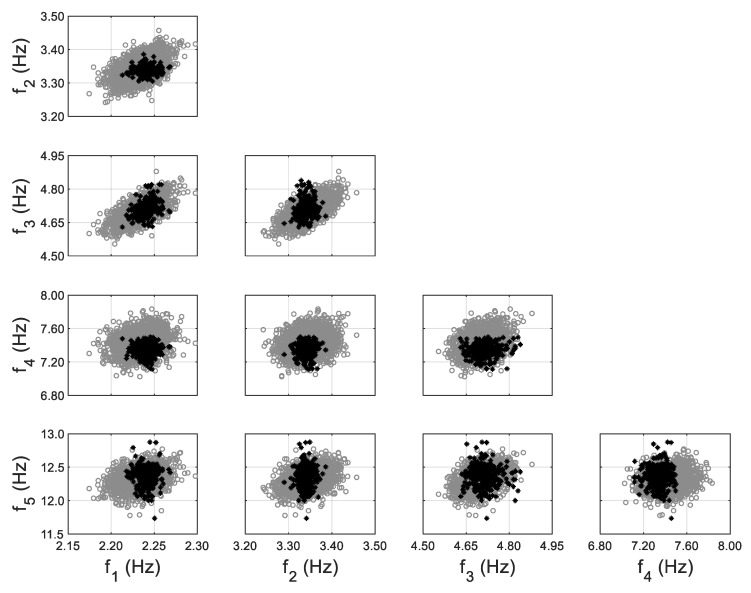
Model-predicted (gray) vs. identified (black) natural frequencies considering non-zero-mean error function for 5th mode, with the inclusion of error function.

The model updating process using the non-zero-mean error function follows exactly the same procedure as the previous zero-mean approach, except that the estimated ${\hat{\mu }}_{e}$ in Equation (11) is included, similar to past numerical studies [57,58]. The comparisons of model-predicted natural frequencies and their identified counterparts are shown in Figure 10, using non-zero-mean error function for fifth mode. It can be observed that the bias between two clouds (gray vs. black) is eliminated, and the predictions have a comparable uncertainty for fifth mode as the identified values. The non-zero error mean ${\hat{\mu }}_{e}$ denotes the residual prediction bias, and it should be considered/propagated into response predictions to provide more accurate and tighter confidence bounds. The MAP values of hyperparameters and error covariance for both cases (zero-mean vs. non-zero-mean error function) are reported in Table 2, together with the evaluated error mean for the 5th mode (last row). It can be seen that all hyperparameters and error covariance values have been kept almost the same except for ${\hat{\sigma }}_{\lambda_{5}}$ between zero-mean and non-zero-mean error function cases. The value of ${\hat{\sigma }}_{\lambda_{5}}$ has been significantly reduced from 0.188 to 0.021, which provides significantly tighter response predictions for the 5th mode, as can be seen in Figure 9 and Figure 10.

#### 3.2.3. Model Predictions for Future Building Conditions

**Figure 11:**
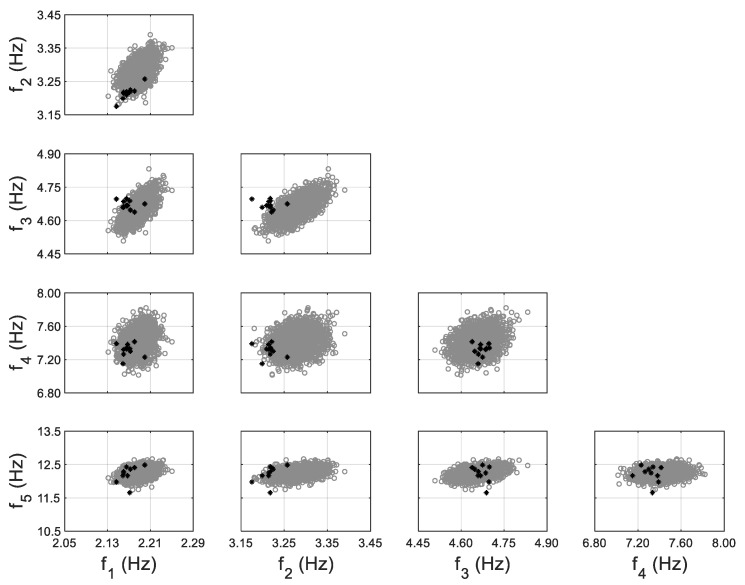
Model-predicted (with the propagation of error function) (gray) vs. identified (black) natural frequencies of the 10-story building at moderate damage state, considering non-zero-mean error function for 5th mode.

**Figure 12:**
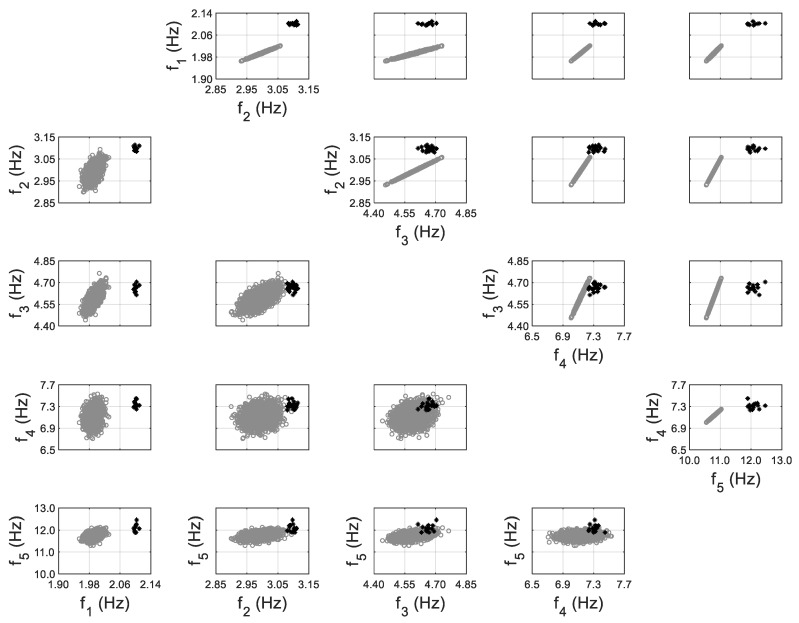
Model-predicted (gray) vs. identified (black) natural frequencies of the 10-story building at severe damage state, considering non-zero-mean error function for 5th mode (upper triangular subplots denote predictions without error function considered; lower triangular subplots denote predictions with propagation of error function).

Calibrated models are often used to predict structural response at different states under future loading for which the model is not calibrated. This is referred to as ‘model extrapolation’. In this application, the calibrated model using non-zero-mean error function is used to predict modal parameters of the building at future damage states. During the dynamic tests of the building, two damage states were introduced by sequentially removing six infill walls. The moderate damage state is defined when four exterior walls in the third story were removed, while the severe damage state denotes the removal of two additional walls in the second story, as shown in Figure 8b. The building condition before wall removals is referred to as the reference state for which the model updating is performed. The ambient vibration of the building was also recorded at its damage states, with 12 and 22 sets of modal parameters identified for moderate and severe damage states, respectively. These identified values are compared with model-predicted natural frequencies in Figure 11 for moderate damage state and Figure 12 for severe damage state. Note that the wall removals are considered in the calibrated model for predictions at the damage states. An acceptable agreement is observed between model-predictions and identified values for the moderate damage state. However, for the severe damage state, the identified values fall out of high probability prediction regions, especially for modes 1 and 2. This can be due to the fact that the estimated error function distribution ($\mu_{e}$ and $\Sigma_{e}$) does not hold when the building state changes significantly (e.g., wall removals at both stories), but acceptable predictions are still obtained for smaller changes in the building state (e.g., removal of walls in just the third story).

### 3.3. Application 3: Two-Story RC Building in El Centro, California

#### 3.3.1. Test Structure and Measured Data

**Figure 13:**
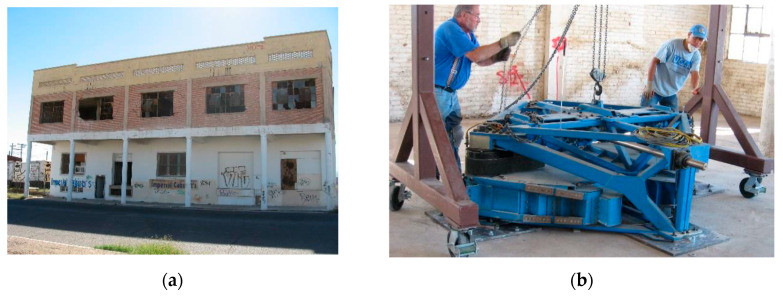
(**a**) North view of the structure; (**b**) Eccentric mass shaker.

The third application is a two-story RC building located in El Centro, California, shown in Figure 13a [6,56,69,70]. The building was severely damaged by past earthquakes and left uninhabited, which provided an opportunity to perform wall removals and destructive shaking tests on this full-scale RC building. The structure consisted of six RC frames in north-south direction which were connected by beams and slabs, and peripheral masonry panels on both stories. Ambient and forced vibration tests were performed on the structure, and acceleration responses were recorded using 15 tri-axial accelerometers located at four corners and the center on the first story, second story and roof. The shaking tests were performed using an eccentric mass shaker (Figure 13b), located on the second floor. The shaker was used to generate harmonic shaking forces in either north-south or east-west directions, and in the form of since-sweep or sine step. 

**Figure 14:**
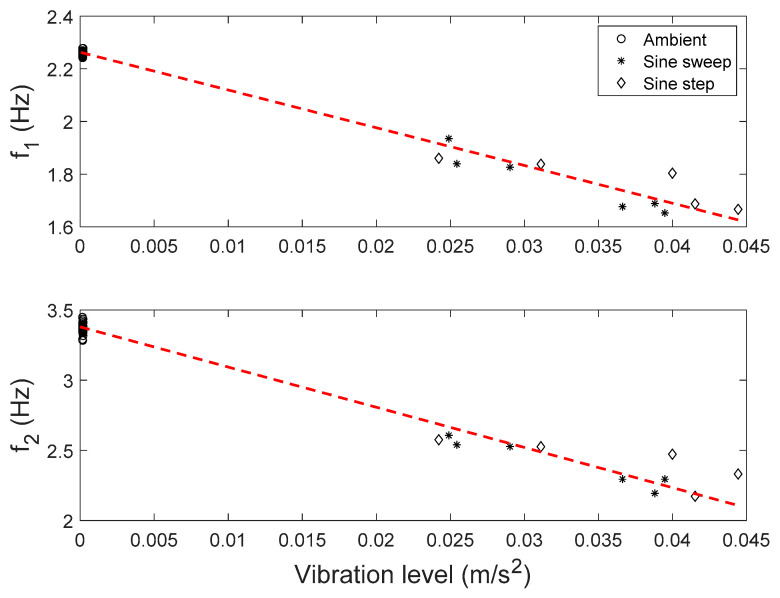
Identified natural frequencies vs. vibration levels.

Modal parameters of the building are identified from ambient and forced vibration data. The first two vibration modes of the building are identified for a total of 30 datasets from ambient vibration, and 11 datasets from forced vibration. The identified natural frequencies are plotted against vibration levels in Figure 14. A clear trend (shown as dash lines) can be observed between natural frequencies and vibration levels. This is due to the reduction of effective stiffness in cracked concrete and masonry elements when the cracks open during forced vibration tests. This reduction is found to be only temporal/transient, and the effective structural stiffness recovers to its original state after the shaking tests. The identified natural frequencies remain unchanged using ambient vibration data, before and after the shaking tests [71]. 

#### 3.3.2. Hierarchical Bayesian Modeling of Stiffness-Amplitude Relationship

The trend between natural frequencies and vibration amplitude is modeled as a linear relationship between stiffness mean and vibration levels.

(12) $$\mu_{\theta }(\varepsilon_{t})=a+b\varepsilon_{t},$$

where **a** and **b** are new higher-level hyperparameters which represent the intercept and slope of the linear relationship between stiffness mean $\mu_{\theta }$ and vibration level $\varepsilon_{t}$. The vibration level is defined as the average RMS of acceleration measurements. Based on a sensitivity analysis, the structure is divided into five substructures with five stiffness parameters, i.e., west ($\theta_{1}$), east ($\theta_{2}$), north ($\theta_{3}$) and south ($\theta_{4}$) walls of the 2nd story, and walls of the 1st story and all columns in the building ($\theta_{5}$). Therefore, **a** and **b** are 5 × 1 vectors. The stiffness variance $\Sigma_{\theta }$ is assumed to be a constant diagonal matrix, as the variance of identified natural frequencies does not appear to be affected by different vibration levels (Figure 14). The error function is assumed to follow a zero-mean Gaussian distribution with a diagonal covariance matrix $\Sigma_{e}$. The hierarchical Bayesian method is applied to estimate the hyperparameters (**a**, **b**, and $\Sigma_{\theta }$) and the error covariance matrix ($\Sigma_{e}$). In this application, the MAPs of updating parameters are computed using the simplified approach reviewed in Section 2.4.2, and then, MH within Gibbs sampling method was employed to sample the joint posterior PDF using the MAPs as starting point, to save computational efforts. This two-step sampling approach can provide the MAP values and the parameters estimation uncertainties. The MAP values of hyperparameters and error covariance using the simplified approach are reported in Table 3. It can be seen that all values of $\hat{b}$ which represents the slope in Figure 14 have negative values, as expected, while the values of $\hat{a}$ represent the mean of structural stiffness at zero vibration level.

#### 3.3.3. Time History Response Predictions

**Figure 15:**
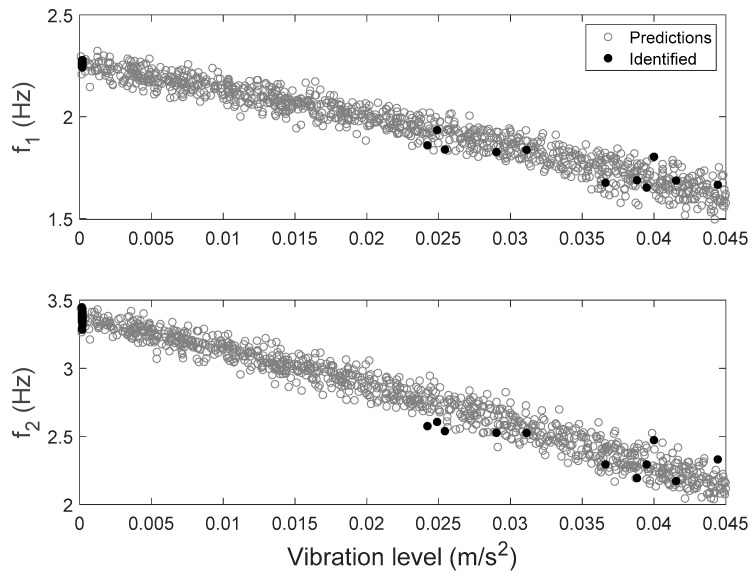
Natural frequencies predictions with their identified counterparts [56].

**Figure 16:**
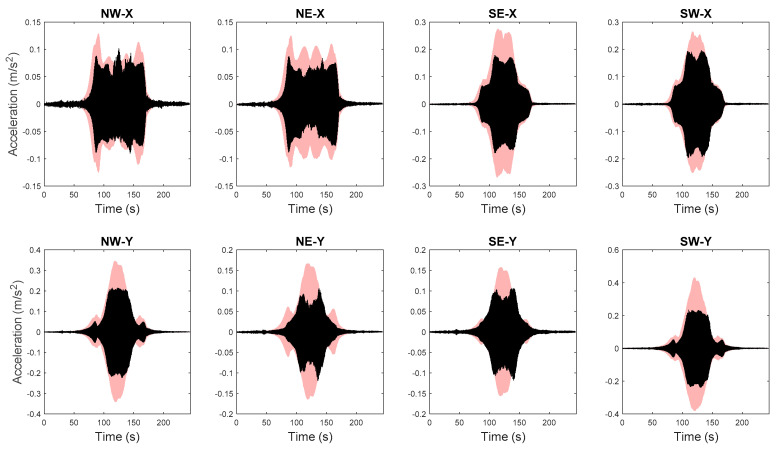
Acceleration time history predictions and measured counterparts (light pink areas refer to 95% quantiles and black lines denote measured data) [56].

**Figure 17:**
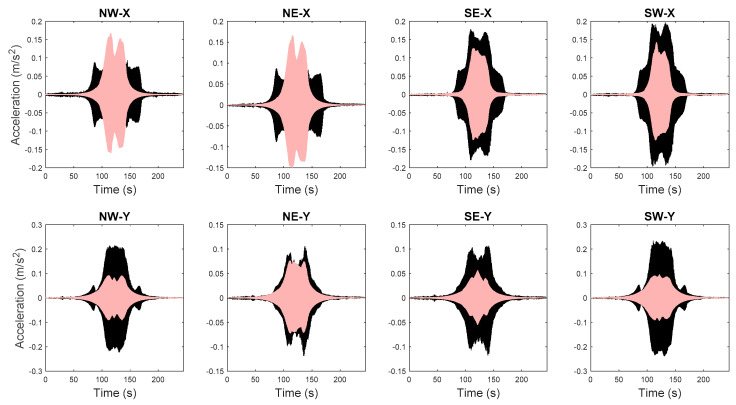
Acceleration time history predictions using ambient vibration level and measured counterparts (light pink areas refer to 95% quantiles and black lines denote measured data) [56].

The FE model of the building is created in OpenSees [72], an open source FE analysis software. The masonry walls are modeled as struts, and floor slabs are assumed to be rigid in-plane. The model is calibrated through the proposed hierarchical framework using identified modal parameters, and is then used to generate 1000 predictions of natural frequencies with vibration levels uniformly distributed within the test range. Structural parameters variability and error function uncertainty are both propagated into these predictions. The predicted natural frequencies are compared with their identified counterparts in Figure 15. It can be seen that model predictions follow the same decreasing trend and cover the scattering uncertainty of the identified values. Acceleration response time histories to a shaker excitation are also predicted from the calibrated model, using the modal superposition method, based on the two modes used in model updating. The comparisons between acceleration time history predictions and their measured counterparts are shown in Figure 16 for a shaker test. It is observed that model predictions have good agreements with measured data, and measurements generally fall in the 95% confidence intervals. The predictions have similar but slightly larger peak amplitudes compared to the measurements. The calibrated model is also used to predict acceleration time histories without considering the stiffness-amplitude relationship, and the comparisons are shown in Figure 17. It is seen that, in this case, the confidence bounds do not fully contain the measurements. A comparison of these two cases demonstrates the importance of accounting for vibration levels in response predictions, when the excitation levels significantly affect the dynamic behavior of the structure. 

## 4. Summary and Conclusions

Model updating applications to civil structures are influenced by different sources of uncertainties, and two of the most important sources are changing ambient/environmental conditions and modeling errors. The reviewed hierarchical Bayesian framework has the capability of explicitly estimating these uncertainties and embedding them in structural parameters variability and error functions. The effects of measurement noise, inherent variability of structural properties and modeling errors are modeled as Gaussian distribution characterized by stiffness hyperparameters $\mu_{\theta }$ and $\Sigma_{\theta }$. Additional higher-level hyperparameters can be introduced to account for other influential factors, e.g., temperatures and excitation levels, to reduce the stiffness variability $\Sigma_{\theta }$. The error function is modeled as Gaussian distribution to quantify the remaining misfit and uncertainty of prediction error, with its own distribution parameters $\mu_{e}$ and $\Sigma_{e}$. The estimated stiffness hyperparameters and error function distribution can then be propagated into response predictions to provide robust and realistic confidence bounds. Three full-scale real-world applications are reviewed in this paper, and the findings are summarized below:

(1) In the application of the Dowling Hall footbridge, different information levels are considered for stiffness hyperparameters formulation and compared for their performance. It is found that the stiffness variability is reduced when information about ambient temperatures and excitation amplitudes is considered, thus providing tighter confidence intervals for model-predictions. In this study, error function must be included in model predictions to provide realistic confidence bounds.
(2) In the application of the 10-story building, the effects of error bias are studied, and it is found that more accurate and tighter prediction bounds are obtained when the error bias $\mu_{e}$ of the fifth mode (which was observed to be biased) is considered. It is also found that the estimated error distribution may not be valid outside the calibration range. Therefore, special precautions should be taken when the calibrated model is used for extrapolation.
(3) In the application of the two-story building, the stiffness mean vector is assumed to have a linear relationship with the vibration levels. Accurate predictions are observed for modal parameters and acceleration time histories using the calibrated model when the stiffness-amplitude dependency is explicitly considered, while inaccurate results are observed when this correlation is neglected.

The reviewed applications highlight the benefits of hierarchical Bayesian model updating compared to traditional deterministic and classical Bayesian model updating methods, including the following.

(a) The hierarchical framework is capable of quantifying structural inherent variability and modeling errors, through postulating probability distributions for structural parameters, and estimating the hyperparameters of these distributions. The estimated structural parameters uncertainty would converge to a constant variation level depending on MAP values of hyperparameters, while parameters uncertainty using classical Bayesian methods is reduced infinitely with more data.
(b) More accurate and robust prediction bounds are achieved by hierarchical framework through propagating of parameters variability and error function. This is often more valuable than just obtaining an accurate prediction fit with measurements. Moreover, more reasonable prediction bounds are obtained compared to the classical Bayesian approach, even when only propagating parameters variability, which is especially useful for predictions of unobserved quantities where error function estimate is not available.
(c) Different relationships and factors that contribute to structural parameters uncertainty can be embedded into the hyperparameters, e.g., ambient temperature and excitation amplitude, in the hierarchical framework, which would reduce the parameters variability and provide tighter prediction confidence bounds.
(d) The hierarchical framework is capable of quantifying the residual prediction errors, by estimating the distribution parameters of error function. The inclusion and propagation of error function into response predictions is important and necessary in some cases when a significant amount of uncertainties is retained in the error function, e.g., Applications 1–3. Considering non-zero-mean error distribution in the presence of error bias reduces the error covariance matrix, thus providing tighter confidence bounds.

## Figures and Tables

**Table 1:** Statistics of hyperparameters and error covariance for information level 3.

	*Q*	*S*	*R*	ϒ	τ	*Y*	σ	$\log(\sigma_{e})$
Mean	1.013	−0.005	0.198	−1.101	3.147	−0.013	0.040	−4.254
Standard deviation	0.0030	0.0001	0.0027	0.0513	0.0861	0.0004	0.0002	0.0012

**Table 2:** MAP values of hyperparameters and error covariance for zero-mean and non-zero-mean error function cases (estimated error mean/bias is shown in last row).

		Zero-Mean Error Function	Non-Zero-Mean Error Function for 5th Mode
Hyperparameters	${\hat{\mu }}_{\theta_{1}}$	0.995	0.995
	${\hat{\mu }}_{\theta_{2}}$	1.132	1.129
	${\hat{\sigma }}_{\theta_{1}}$	0.021	0.021
	${\hat{\sigma }}_{\theta_{2}}$	0.015	0.014
Error covariance (for eigenvalues)	${\hat{\sigma }}_{\lambda_{1}}$	0.010	0.011
	${\hat{\sigma }}_{\lambda_{2}}$	0.014	0.014
	${\hat{\sigma }}_{\lambda_{3}}$	0.007	0.007
	${\hat{\sigma }}_{\lambda_{4}}$	0.034	0.034
	${\hat{\sigma }}_{\lambda_{5}}$	**0.188**	**0.021**
Error mean	${\hat{\mu }}_{\lambda_{5}}$	0	−0.185

**Table 3:** MAP values of hyperparameters and error covariance using simplified approach.

		$\hat{a}$	$\hat{b}$	${\hat{\sigma }}_{\theta }$			${\hat{\sigma }}_{e}\;(\%)$
Hyperparameters	$\left(\theta_{1}\right)$	0.11	−2.41	0.011	Error covariance	$\left(\lambda_{1}\right)$	0.02
	$\left(\theta_{2}\right)$	0.85	−4.68	0.008		$\left(\lambda_{2}\right)$	0.98
	$\left(\theta_{3}\right)$	0.08	−0.44	0.010		$\left(\Phi_{1}\right)$	1.32
	$\left(\theta_{4}\right)$	0.40	−4.91	0.017		$\left(\Phi_{2}\right)$	1.98
	$\left(\theta_{5}\right)$	1.82	−22.66	0.065			

## Supplementary Materials

The experimental data of the Dowling Hall footbridge in Application 1 is available online at https://engineering.tufts.edu/cee/shm/research_BM_continuousMonitoring.asp#weeks. The experimental data of the 10-story RC building in Utica, New York in Application 2 are available online at https://www.designsafe-ci.org/data/browser/public/designsafe.storage.published/PRJ-1710/#details-9050751670063796711-242ac11c-0001-012.
